# Secure aggregation of sufficiently many private inputs

**DOI:** 10.3389/fdata.2025.1638307

**Published:** 2025-09-10

**Authors:** Thijs Veugen, Gabriele Spini, Frank Muller

**Affiliations:** ^1^Unit ICT, Strategy and Policy, TNO, The Hague, Netherlands; ^2^Department of Semantics, Cybersecurity and Services, University of Twente, Enschede, Netherlands; ^3^Department of Cyber Security, Austrian Institute of Technology (AIT), Vienna, Austria

**Keywords:** secure multi-party computation, secure aggregation, Shamir secret sharing, cyber threat intelligence, security model

## Abstract

Secure aggregation of distributed inputs is a well-studied problem. In this study, anonymity of inputs is achieved by assuring a minimal quota before publishing the outcome. We design and implement an efficient cryptographic protocol that mitigates the most important security risks and show its application in the cyber threat intelligence (CTI) domain. Our approach allows for generic aggregation and quota functions. With 20 inputs from different parties, we can do three secure and anonymous aggregations per second, and in a CTI community of 100 partners, 10, 000 aggregations could be performed during one night.

## 1 Introduction

The secure aggregation of data is a common problem when data from different sources need to be combined. Although single inputs are often sensitive, either from a privacy or a commercial point of view, their aggregation often no longer is. This is especially true when sufficiently many inputs have been added, such that the outcome does not leak information about the single inputs anymore.

In this study, we describe a cryptographic solution for this problem that is applicable in many different domains. For example, when sensor information needs to be accumulated in a network base station to measure traffic flows. Also in smart metering when energy consumption of a community needs to be aggregated over different households. Other examples are the combination of data from telemetry devices in medical research, and more general population polling and statistical surveys.

We have implemented our solution for usage in Cyber Threat Intelligence (CTI) where organizations combine sightings of indicators of compromise to build a joined CTI dashboard that provides the latest information of relevant cyber threats (MISP, 2022).

### 1.1 Problem statement and contribution

We state the aggregation problem in a more formal way. There are *n* parties, each having a sensitive input *x*_*i*_, and one aggregator that is securely adding up all inputs. Inputs have integer values, cannot be negative, and consist of at most *m* bits, i.e., $x_{i}\in \left\{0,1,\ldots 2^{m}-1\right\}$. If party *i* does not have a contribution, then it sets *x*_*i*_ = 0, i.e., we want all parties to provide an input, because we do not want to reveal which parties did actually contribute, as this information could be sensitive. In the CTI use case, parties set their input to zero when the indicator of compromise has not been sighted during a certain time period. The requirements that have been derived with the CTI use case in mind are as follows:

1. Individual inputs (some of them could be zero) should remain private.
2. The sum should only be published by the aggregator, if sufficiently many (at least threshold *k*) positive inputs have been added.
3. Malicious parties could collude with each other, or with the (potentially malicious) aggregator. Our solution should be secure with honest majority.
4. The *n* parties cannot communicate directly (not scalable) and should use the aggregator for that.
5. The solution should be efficient and scalable for large *n*.

We have designed and implemented a cryptographic protocol based on Shamir secret sharing that fulfills these requirements. The protocol is secure in the semi-honest model and mitigates a couple of additional security risks. In particular, we want to avoid parties pretending to have a contribution when they actually do not. That would allow them to learn inputs from other parties (by maliciously achieving the contribution threshold *k*) without disturbing correctness of the outcome. For many applications, it is important that the output is correct, because, e.g., in the CTI use case, the outcomes are used by the aggregator (CTI community manager) to present statistics on current important threats. Although we use an aggregator to publish the results, which is required for certain applications, our solutions work just as well without an aggregator. In that case, the sum is revealed to all parties instead of one aggregator.

Even though our solution fulfills all practical requirements mentioned, at least from a theoretical perspective it is interesting to investigate how an actively secure solution would look like. Therefore, we sketch in Appendix A how to extend the protocol such that it becomes secure in the malicious model. That means that parties are no longer assumed to follow the rules of the protocol and may try to actively learn private inputs from other parties.

### 1.2 Related work

There is a lot of related work on the area of secure aggregation. However, they do not take into account that the sum can only be revealed if there are sufficiently many contributions, which is the main part of our contribution. The metric we use for preserving privacy is similar to *k*-anonymity, parameter *k* being our threshold.

One exception is Bonawitz et al. (2017), who present secure aggregation solutions (both actively and passively secure ones) for dynamic environments, where parties are not required to submit an input. The sum is revealed when sufficiently many inputs have been received. However, they do not hide the identity of contributing users. On the other hand, our solution is not suitable for dynamic environments: Users might come and go over different aggregations but not during one secure aggregation protocol.

A similar problem was studied by the seminal paper by Shi et al. (2011) on privacy-preserving aggregation. The secure aggregation problem is also a well-studied problem within federated learning, where local model parameters need to be aggregated securely (Liu et al., 2022).

Xu et al. (2020) present a method to securely compute arbitrary aggregation functions of multiple inputs using lightweight cryptography. Given that maximally *k* participants can collude, they achieve *n* − *k* source anonymity, i.e., the identity of only one of the *n* − *k* non-colliders is leaked to the aggregator. We avoid that leakage, and more importantly, we check whether sufficiently many parties have contributed. We also facilitate arbitrary aggregation functions and different contribution thresholds, by going through the bits (see Section 2).

A recent solution by Bozdemir et al. (2024) solves the problem by using threshold homomorphic encryption and additive secret sharing with two aggregators. Instead of one aggregator, they assume two aggregators that are not allowed to collude. The inputs are easily added with homomorphic encryption, and the threshold question is resolved by secure two-party computations, using additive secret sharing, between the two aggregators. The main problem with this solution is the risk of collusion between the two aggregators, who jointly act as a trusted third party.

## 2 Sufficiently many positive inputs

In the semi-honest model, an easy solution is to have each party compute the additional indicator *n*_*i*_, which is one, if *x*_*i*_ > 0, and zero, otherwise, and then securely aggregating the *n*_*i*_. We use the Iverson bracket notation [.] for binary indicator *n*_*i*_.

1. Each party *i* inputs *x*_*i*_ and *n*_*i*_, such that *n*_*i*_ = [*x*_*i*_ > 0].
2. The parties securely compute and reveal $\underset{i}{\sum }n_{i}$.
3. If $\underset{i}{\sum }n_{i}\geq k$, then the parties securely compute and reveal $\underset{i}{\sum }x_{i}$.

However, we want to avoid parties misbehaving by setting *n*_*i*_ = 1 (or even a larger positive value) in case *x*_*i*_ = 0 to learn other sensitive inputs (through their revealed sum).

The secure addition of inputs can be easily implemented with (Shamir) secret sharing or additively homomorphic encryption. The challenge is to find an efficient solution for counting the number of positive inputs. To be able to check consistency between the inputs *x*_*i*_ and the non-zero counters *n*_*i*_, we split the inputs into bits:


$$x_{i}=\overset{m-1}{\underset{j=0}{\sum }}2^{j}x_{i,j},$$

such that the non-zero counter *n*_*i*_ can be computed by


$$n_{i}=1-\overset{m-1}{\underset{j=0}{\prod }}\left(1-x_{i,j}\right).$$

Although entering the inputs as bits is not the only way to assure consistency with the non-zero indicator^1^, it is a convenient way for computing additional constraints that need to be fulfilled before the sum, or another function of the inputs, is allowed to be computed.

### 2.1 Multiplication

To compute the non-zero indicators *n*_*i*_, we need to be able to compute the product of *m* secret (binary) values. As homomorphic encryption takes quite some computational and communication effort, we prefer to arrange this with secret sharing techniques. We choose Shamir secret sharing, which is known to efficiently compute secure inner products. We set secret-sharing threshold *t* = ⌊(*n* − 1)/2⌋ (*t* is the degree of the secret-sharing polynomial), such that we need *t* + 1 out of *n* shares to reconstruct a secret, and the eventual solution will be secure with honest majority (see third requirement).

A Shamir secret-sharing with threshold *t* of secret *x* is denoted by 〈*x*〉_*t*_. A well-known way to compute and reveal the inner product of two secret vectors (*x*_1_, …, *x*_*k*_) and (*y*_1_, …, *y*_*k*_), given their secret sharings with threshold *t*, is as follows (de Hoogh, 2012, Protocol 4.10):

1. The parties locally multiply their shares of *x*_*i*_ and *y*_*i*_ to obtain a secret-sharing 〈*x*_*i*_ · *y*_*i*_〉2_*t*_.
2. The parties locally generate a zero secret-sharing 〈0〉_2*t*_ (see Appendix B).
3. They reconstruct the inner product $\underset{i}{\sum }x_{i}\cdot y_{i}$ from ${\langle 0+\underset{i}{\sum }x_{i}\cdot y_{i}\rangle }_{2t}$ by Lagrange interpolation.

The outcome is revealed in step 3 by combining 2*t* + 1 ≤ *n* shares of the inner product. The additional secret-sharing of zero is needed for security reasons and can be generated without communication through pseudo-random functions and replicated secret sharing (de Hoogh, 2012, Protocol 4.7) (see Appendix B for the main ideas).

Therefore, when *m* = 2, we can compute and reveal the number of non-zeros $\sum_{i}n_{i}$ with this secure inner product protocol, because then *n*_*i*_ = 1 − (1 − *x*_*i*,0_) · (1 − *x*_*i*,1_). However, for larger *m*, we have a problem. We could use a secure multiplication protocol without revealing the product, but this would require additional communication between the parties, which we want to avoid for scalability reasons.

Therefore, we introduce $y_{i}=\sum_{j=0}^{m-1}x_{i,j}$, being the sum of the bits of input *x*_*i*_. We know that *x*_*i*_ = 0, if and only if, *y*_*i*_ = 0, and more importantly, the size of *y*_*i*_ is only log_2_*m* instead of *m*. With two variables, *x*_*i*_ and *y*_*i*_, we can compute [*y*_*i*_ > 0], if *y*_*i*_ has at most two bits (and is at most three), i.e., *x*_*i*_ has at most three bits (and is at most seven). And by adding another variable *z*_*i*_ (of two bits), we can even extend that further to $x_{i}\leq 2^{7}-1=127$, such that


$$n_{i}=\left[x_{i}>0\right]=\left[y_{i}>0\right]=\left[z_{i}>0\right]=1-\left(1-z_{i,0}\right)\cdot \left(1-z_{i,1}\right).$$

Given the *z*_*i*_, we can compute $\underset{i}{\sum }n_{i}$ by revealing one secure inner product (see Equation 1 below). If the inputs *x*_*i*_ are at most 2^7^ − 1 = 127, we can suffice with having only three variables *x*_*i*_, *y*_*i*_, and *z*_*i*_ per input. With one more layer, we can even cope with inputs *x*_*i*_ of size 2^127^ − 1. To avoid forgery with the *n*_*i*_, we have to check whether the bits of *x*, *y*, up to *z* are consistent with each other.

### 2.2 Consistency checks

Although formally not required for passive security, we want to avoid a number of straightforward attacks, such that party *i* cannot set *n*_*i*_ = 1 when *x*_*i*_ = 0. We consider two types of checks, to be performed without revealing the inputs:

(a) Check the consistency between *x*_*i*_, *y*_*i*_, and eventually *z*_*i*_. E.g., to check the consistency between *x*_*i*_ and *y*_*i*_, we need to verify that
$$\underset{j}{\sum }x_{i,j}=\underset{j}{\sum }2^{j}y_{i,j}.$$
  This is done by local computation of 〈α_*i*_〉_*t*_, where $\alpha_{i}=\underset{j}{\sum }x_{i,j}-\underset{j}{\sum }2^{j}y_{i,j}$, revealing α_*i*_ (through broadcasting *t* + 1 shares) and checking α_*i*_ = 0.
(b) We need to assure that all supposed bits (*x*_*i,j*_, *y*_*i,j*_, up to *z*_*i,j*_) are actually bits. E.g., to check the *j*-th bit of *x*_*i*_, we need to verify that
$$x_{i.j}\cdot \left(1-x_{i,j}\right)=0,$$
  which can be done with the secure inner product protocol from Section 2.1: locally compute 〈β_*i,j*_〉2_*t*_ = 〈*x*_*i,j*_〉_*t*_ · 〈1 − *x*_*i,j*_〉_*t*_, add a fresh 〈0〉_2*t*_, reveal β_*i,j*_ + 0 (through broadcasting 2*t* + 1 shares), and check β_*i,j*_ = 0.

If one of the α_*i*_ or β_*i,j*_ is not zero, the corresponding input is not valid.

Since each separate check requires additional communication, we found a way to combine them all into two zero-check protocols (one for each of the two types) to reduce communication efforts (in line with requirement five). The idea is that each check value α_*i*_, e.g., $\alpha_{i}=\underset{j}{\sum }x_{i,j}-\underset{j}{\sum }2^{j}y_{i,j}$, is multiplied with a random weight *w*_*i*_, such that we only need to check whether


$$\underset{i}{\sum }\alpha_{i}\cdot w_{i}=0.$$

It is important that the weights *w*_*i*_ are not revealed before the input shares of *x*_*i*_, *y*_*i*_, up to *z*_*i*_, have been distributed among the players, such that they cannot influence the check outcome. The probability of forging the combined zero-check will be 1/*p*, where *p* is the (large) prime of our finite field. A way to generate the weights is:

1. The parties locally generate the secret sharing 〈*r*〉_*t*_ of a random secret number *r*.
2. They reveal *r* (through broadcasting *t* + 1 shares) and use it for generating random weights. Each party computes weight *w*_*i*_ as follows:
  (a) ω←PRF(*r, i*) { Use a pseudorandom function to create a fresh random number ω. }
  (b) Set *w*_*i*_ ← 1 + (ω mod (*p* − 1)) { *w*_*i*_ ∈_*R*_{1, 2, …, *p* − 1} }

The input shares should be distributed before *r* is revealed. The secret-sharing of *r* in step 1 can be generated without communication through pseudo-random functions (de Hoogh, 2012, Protocol 4.6) (similar to Appendix B). When *r* is revealed, each player can generate the weights locally.

We can combine all first type checks, being secret sharings 〈α_*i*_〉_*t*_ of degree *t* into one, and similarly all second type checks, being secret sharings 〈β_*i,j*_〉2_*t*_ of degree 2*t*, although one zero-sharing 〈0〉_2*t*_ needs to be added there (as before, see Appendix B how to generate it) for security reasons.

## 3 Overview of passively secure solution

For completeness, we present the entire cryptographic protocol for the passively secure model that assures consistency between the inputs *x*_*i*_ and the non-zero indicators *n*_*i*_. All communication between the parties goes via the aggregator (according to requirement four). We assume a Public Key Infrastructure (PKI) is used to enable confidential and authenticated communication. The parties (excluding the aggregator) in particular create pairwise-secure communication channels that are private and authenticated.

**Table:** 

Inputs	$x_{i}\in \left\{0,1,\ldots ,2^{m}-1\right\}$, 1 ≤ *i* ≤ *n*
First output Second output	$\overset{n}{\underset{i=1}{\sum }}n_{i}$, such that *n*_*i*_ = [*x*_*i*_ > 0] $\overset{n}{\underset{i=1}{\sum }}x_{i}$
Condition	The second output is only computed if $\overset{n}{\underset{i=1}{\sum }}n_{i}\geq k$

1. Each party *i* computes the bits *x*_*i,j*_, 0 ≤ *i* < *m*, of its input *x*_*i*_, and consequently the bits *y*_*i,j*_ of $\underset{j}{\sum }x_{i,j}$, etc. up to the bits *z*_*i,j*_ (see Section 2.1).
2. Each party *i* generates Shamir secret-sharings 〈*x*_*i,j*_〉_*t*_, 〈*y*_*i,j*_〉_*t*_, up to 〈*z*_*i,j*_〉_*t*_, 0 ≤ *j* < *m* and sends the shares through the aggregator to the proper parties.
3. The parties generate the weights that are needed for all consistency checks (see Section 2.2).
4. The parties check consistency of all inputs by revealing two combined checks, one for each consistency type (see Section 2.2).
  (a) Locally compute ${\langle \underset{i}{\sum }\alpha_{i}\cdot w_{i}\rangle }_{t}$, reveal it through broadcasting *t* + 1 shares, and verify it is zero.
  (b) Locally compute ${\langle \underset{i}{\sum }\beta_{i}\cdot w_{i}^{\prime }\rangle }_{2t}+{\langle 0\rangle }_{2t}$, reveal it through broadcasting 2*t* + 1 shares, and verify it is zero.
  If a check fails, the protocol aborts.
5. The parties locally compute their share of the total number of non-zero inputs and reveal
(1) $$\begin{array}{l} \underset{i}{\sum }n_{i}=n-\underset{i}{\sum }\left(1-z_{i,0}\right)\cdot \left(1-z_{i,1}\right) \end{array}$$
  using the secure inner product protocol from Subsection 2.1.
6. If there are not sufficiently many (at least *k*) non-zero inputs, the protocol aborts.
7. The parties locally compute (their share of) a secret-sharing of the sum of all inputs
$$\underset{i}{\sum }\overset{m-1}{\underset{j=0}{\sum }}2^{j}{\langle x_{i,j}\rangle }_{t},$$
  and reveal it by broadcasting *t* + 1 shares.

Because we check the consistency between inputs and non-zero indicators, parties cannot set *n*_*i*_ = 1, if *x*_*i*_ = 0. We implemented this protocol; for details and performance, see Section 5.

## 4 Security evaluation

The protocol is secure with honest majority, achieving statistical security (forgery succeeds with probability 1/*p*) with abort. This easily follows because we use standard subprotocols for Shamir secret sharing that are known to be secure in the semi-honest model (de Hoogh, 2012).

As explained at the start of Section 2, our solution is more complex than strictly necessary for this security model, because we want to avoid a couple of easy attacks that are attractive within a CTI community, and potential other use cases. To clearly explain which attacks have been mitigated, and which have not, we evaluate the security of our passively secure protocol from Section 3 and list most attacks an adversary could attempt, and the reason why they fail, or must be regarded out-of-scope.

We assume any outside attacker has no access to private PKI keys and will not be able to send authenticated messages or decrypt intercepted messages. Therefore, we restrict the evaluation to inside players that are malicious, including the aggregator.

### 4.1 Malicious players

Here, we consider possible attacks by players, other than the aggregator, not following the rules of the protocol.

1. **Dishonest participants inserting fake inputs other than zero**. Although each input bit is checked, an erroneous input cannot be prevented in any protocol; participants' values must be treated as is. This does not reveal data of honest parties but will result in erroneous output.
2. **A dishonest minority of the participants trying to retrieve honest parties' inputs from the distributed shares**. This part of the solution is even unconditionally secure; its security is information-theoretic, based on Shamir secret sharing. It takes at least ⌈(*n* − 1)/2⌉ players to reconstruct data with Lagrange interpolation.
3. **A dishonest minority sending in zeroes and trying to disguise this by cheating with bits of the** **y** **or** **z** **values** This is not possible because all bits of the *y* and *z* values are jointly checked for consistency with the *x* and *y* values, respectively.
4. **A dishonest minority trying to manipulate the revealing process of the checks to be able to carry out the previous attack anyhow**. This will be detected, *provided that after Lagrange interpolation, it is checked that the contribution of each of the participants is on the fitted polynomial* (see Subsection A.2). If one or more of the contributions are not consistent with the polynomial, the protocol will abort.
5. **After all checks are done, a dishonest minority sending in not the shares of the sum, but a different value**. This is possible, but will be detected, *provided that after Lagrange interpolation, it is checked that the contribution of each of the participants is on the fitted polynomial* (see Subsection A.2). If not, the protocol will abort.

The final two risks can be mitigated quite easily by checking the consistency of all revealed shares. In that case, revealing a secret value requires broadcasting all shares, instead of only *t* + 1. This has not been implemented in our passively secure protocol, although it would only result in a minor increase in communication and computation and not affect the order of complexity.

More sophisticated attacks are possible on certain subprotocols such as random secret generation, multiplication, and zero-check, for which the direct gain of an adversary is less clear. In Appendix A, we sketch a way to mitigate these and make the protocol actively secure with dishonest minority.

### 4.2 Misbehaving aggregator

Here, we consider specific attacks by the aggregator.

1. **The aggregator trying to reveal the output even though the non-zero quota was not reached**. The honest parties will not sent in their shares if the quota was not reached. Even if the aggregator colludes with a dishonest minority and receives all their shares, he cannot retrieve the data, because the Lagrange interpolation will not succeed if there is too little input.
2. **The aggregator trying to reveal the input (shares) of individual parties**. This is not possible, because all shares are encrypted and can only be decrypted by the rightful recipient.
3. **The aggregator enforcing a dishonest majority**. Supposing that the aggregator initializes a protocol run, and controls the admission of (malicious) parties, then a dishonest majority could be created and sensitive inputs could be learned. This is out of scope for our setting, as we assume the number of players *n* is known to all players, and they create shares with threshold *t* that assures security against dishonest minority only.
4. **The aggregator disturbing the communication between players** Because all messages between players are routed through the aggregator, he could easily mess up the communication by delaying or destroying messages. He cannot create authenticated messages himself, but he could replay older messages. The aggregator is not able to change origin or destination of messages because of the PKI. There are two steps where the protocol might abort (see Section 3). If we require all parties to broadcast an “Ok” message before the protocol can proceed, and introduce a session ID, we can circumvent these attacks. A malicious aggregator could then have the protocol abort but not reveal sensitive information.

## 5 Performance

The passively secure protocol has been incorporated into Malware Information Sharing Platform (MISP), a well-known CTI open source platform (MISP, 2022; COSSAS, 2022). The experiments were run on a machine with an Intel Core i7-9850H CPU with six cores and 12 threads, clocking at a base frequency of 2.6GHz and with a maximal turbo frequency of 4.6GHz, equipped with 32GB of RAM, and running Ubuntu 20.04. Aggregator and participants were simulated on this machine as distinct processes.

Our complexity order for growing number of parties, i.e., linear increase in communication and quadratic increase in computation, is identical to Bonawitz et al. (2017), the most similar related work we found. Exploratory tests have indicated that the choice of cryptographic algorithms in the PKI has a negligible effect on the protocol's performance.

### 5.1 Running time

Two shell-scripts have been written, which spawn the desired number of parties, generate random input values, execute the protocol, and measure its running time. For each used parameter set, the process was repeated 10 times, to correct possible fluctuations due to the randomization of the process, or to other tasks requiring usage of the computer resources. The CPU frequency was not manually set to a fixed value. The first script kept the number of participants fixed, while letting the number of Indicators Of Compromise (IOCs) increase; the second one kept the number of IOCs fixed, while letting the number of simulated participants increase.

**Figure 1:**
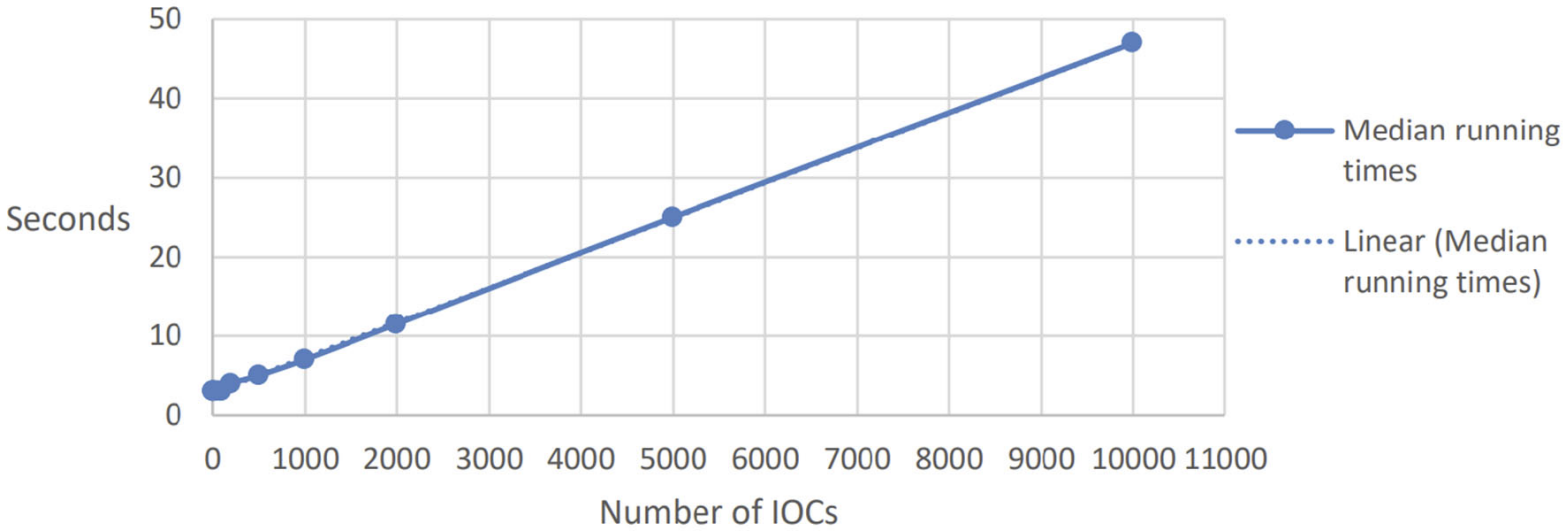
Running time for many IOCs.

**Figure 2:**
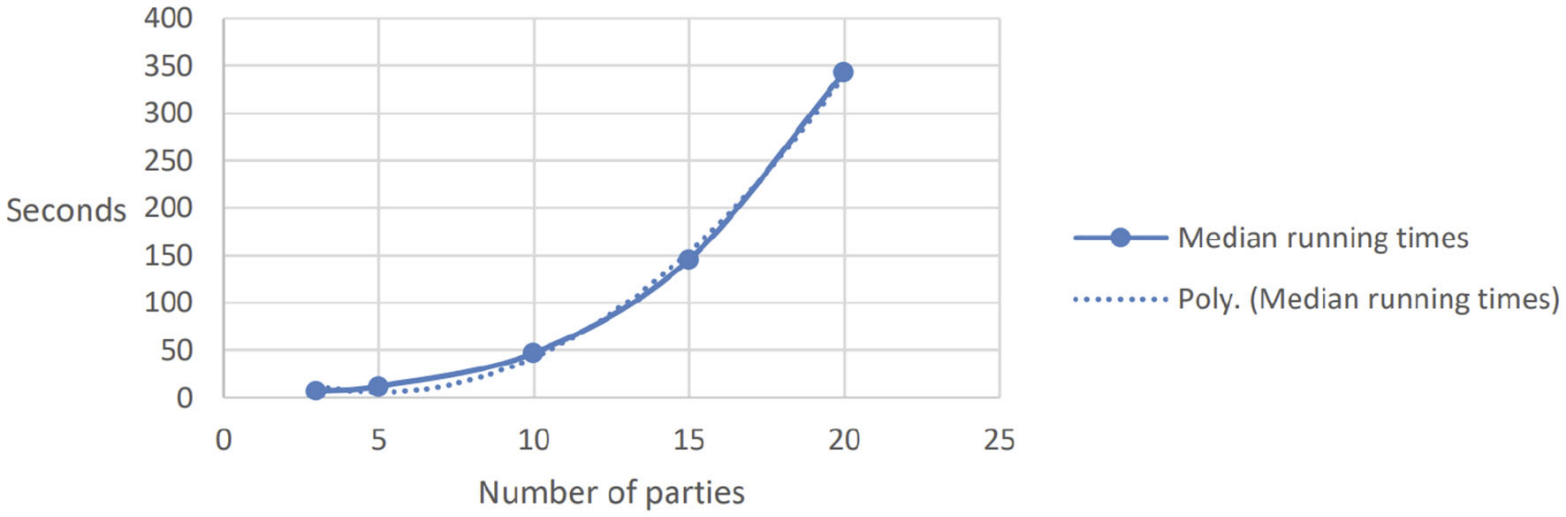
Running time for many parties.

The results of the experiments are reported in Figure 1 (for three parties, one aggregator, and increasing number of IOCs) and Figure 2 (for 1, 000 IOCs and increasing number of participants). We report here the median times, as we believe these can give a more accurate picture compared to average results, since they reduce the impact of outliers; however, the results are quite consistent across the different repetitions of each parameter set, with a standard deviation of at most 0.7.

The number of Indicators of Compromise (IOCs) applies to the CTI use case and represents the number of different aggregates that need to be performed. All these instances can, in theory, be executed in parallel, minimizing the communication overhead. We expected a linear dependency between the number of instances and the running time, which is confirmed by the dashed linear line. We can run roughly 200 secure aggregations per second.

Second, we investigated the scalability for growing number of parties, as shown for 1, 000 aggregations in Figure 2. The performance decreases from 200 aggregations per second for three parties to 1, 000/350 ≈ 3 aggregations for 20 parties. Extrapolating the graph yields 4, 000 s for 100 parties, which means that 10, 000 aggregations with 100 parties would take 40, 000 s which is roughly half a day. In our CTI use case, where typically 10, 000 IOCs are reported each day within a community consisting of 100 partners, these could be computed securely overnight.

If *n* parties join the protocol run, every submitted value will be split into *n* shares. The amount of information a party must process increases linearly with the number of participants. Since we are simulating *n* parties, we would expect a quadratically increasing runtime. However, the polynomial approximation shown in Figure 2 indicates a faster expansion.

This may be due to deficiencies in the code, the limited number of parallel processes, or clogging in the machine's processing unit. In our case, we made use of a CPU with 6 cores and 12 threads, meaning that with more than 12 parties, it is impossible for each simulated party to fully utilize the maximum CPU potential for the entire length of the computation. As such, we believe this more-than-quadratic scalability to be the result of the limitations of the testing set-up, rather than of the solution itself.

### 5.2 Communication

In theory, the number of communication rounds stays the same with increasing number of parties. However, a simulation on a single machine does not suffer from communication delays that will occur in distributed simulations.

Furthermore, when running multiple instances of our protocol, the individual aggregation protocols could, in theory, be run in parallel, hence maintaining the running time constant. However, due to physical limitations in bandwidth (for the time spent sending/receiving values, i.e., the communication overhead) and in processing power (for the local computations to be performed), this would not scale. We therefore decided to simply use a sequential execution of the protocol for each instance.

## 6 Conclusion

Private inputs can be aggregated securely with the help of secret sharing. By going through the input bits, we presented a generic platform for securely computing statistics of sensitive inputs, the result only being revealed under specific conditions that guarantee anonymity of inputs.

To demonstrate this, we developed a protocol with robust security and realistic run-times for communities of 100 participants and 10, 000 instances. Although a full extension to the malicious security model has not (yet) been achieved, we are confident that this is within reach. In this way, we show that secure aggregation can be applied broadly. It will support CTI communities to increase their awareness of cyber risks and improve their resilience.

## Data Availability

The original contributions presented in the study are included in the article/Supplementary material, further inquiries can be directed to the corresponding author.

## Footnotes

^1^One could also verify that *n*_*i*_(1 − *n*_*i*_) = 0, *x*_*i*_(1 − *n*_*i*_) = 0, and *x*_*i*_ + 1 − *n*_*i*_ ≠ 0.
