# Supplementary material for: Secure aggregation of sufficiently many private inputs
Source: Front Big Data. 2025 Sep 10;8:1638307. doi: 10.3389/fdata.2025.1638307 (PMC12457162; doi:10.3389/fdata.2025.1638307)
Supplement: Supplementary file 1 [file Data_Sheet_1.pdf]

Secure aggregation of sufficiently many private inputs - Appendix

Thijs Veugen, Gabriel Spini, Frank Muller

August 27, 2025

References

[1] Shai Halevi, Yuval Ishai, Abhishek Jain, Ilan Komargodski, Amit Sahai, Eylon Yogev, Non-interactive multiparty computation without correlated randomness, Advances in Cryptology – ASIACRYPT 2017: 23rd International Conference on the Theory and Applications of Cryptology and Information Security, Hong Kong, China, December 3-7, 2017, Proceedings, Part III, pages 181 - 211, https://doi.org/10.1007/978-3-319-70700-6_7

[2] Ronald Cramer, Ivan Damgård, Yuval Ishai, Share conversion, pseudorandom secret-sharing and applications to secure computations, TCC 2005, Lecture Notes in Computer Science Theory of Cryptography, pp. 342-362, Springer, 10.1007/978-3-540-30576-7_19

[3] Ivan Damgård, Matthias Fitzi, Eike Kiltz, Jesper Nielsen, and Tomas Toft. Unconditionally secure constant-rounds multi-party computation for equality, comparison, bits and exponentiation. In Shai Halevi and Tal Rabin, editors, Theory of Cryptography, volume 3876 of Lecture Notes in Computer Science, pages 285–304. Springer Berlin / Heidelberg, 2006.

[4] Zuzana Beerliová-Trubíniová and Martin Hirt, Perfectly-Secure MPC with Linear Communication Complexity, TCC 2008, LNCS 4948, pp. 213–230, 2008.

[5] Koji Chida, Daniel Genkin, Koki Hamada, Dai Ikarashi, Ryo Kikuchi, Yehuda Lindell, Ariel Nof, Fast Large-Scale Honest-Majority MPC for Malicious Adversaries, Advances in Cryptology - CRYPTO 2018, Springer, 2018.

[6] Y. Lindell and A. Nof. A Framework for Constructing Fast MPC over Arithmetic Circuits with Malicious Adversaries and an Honest-Majority. In CCS '17: Proceedings of the 2017 ACM SIGSAC Conference on Computer and Communications Security, pages 259 - 276, https://doi.org/10.1145/3133956.31339

[7] Daniel Genkin, Yuval Ishai, Manoj M. Prabhakaran, Amit Sahai,Eran Tromer, Circuits Resilient to Additive Attacks with Applications to Secure Computation, STOC 2014: Proceedings of the forty-sixth annual ACM symposium on Theory of Computing, pp. 495-504, May 2014.

[8] Secure aggregation of cyber threat intelligence (SACTI), MISP, 2022 https://www.misp-project.org/2022/10/27/SACTI_Secure_aggregation_of_cyber_threat_intelligence.htm

[9] SACTI source code, available at Github through COSSAS, 2022, https://github.com/COSSAS/sacti

[10] Sebastiaan de Hoogh, Design of large scale applications of secure multiparty computation: secure linear programming. Phd Thesis, Mathematics and Computer Science, Eindhoven University of Technology, 2012.

[11] Shi, E., Chan, HTH., Rieffel, E., Chow, R. and Song, D., Privacy-Preserving Aggregation of Time-Series Data, the 18th Annual Network & Distributed System Security Symposium (NDSS), San Diego, California, USA, Internet Society, 6-9 February 2011.

[12] Z. Liu, J. Guo, W. Yang, J. Fan, K. -Y. Lam and J. Zhao, Privacy-Preserving Aggregation in Federated Learning: A Survey, in IEEE Transactions on Big Data, doi: 10.1109/TBDATA.2022.3190835. 2022.

[13] Bozdemir, B., Özdemir, B.A., Önen, M. (2024). PRIDA: PRIvacy-Preserving Data Aggregation with Multiple Data Customers. In: Pitropakis, N., Katsikas, S., Furnell, S., Markantonakis, K. (eds) ICT Systems Security and Privacy Protection. SEC 2024. IFIP Advances in Information and Communication Technology, vol 710. Springer, Cham. https://doi.org/10.1007/978-3-031-65175-5_4

[14] Chang Xu, Lvhan Zhang, Liehuang Zhu, Chuan Zhang, Xiaojiang Du, Mohsen Guizani, Kashif Sharif, Aggregate in my way: Privacy-preserving data aggregation without trusted authority in ICN, Future Generation Computer Systems, Volume 111, 2020, Pages 107-116, ISSN 0167-739X, https://doi.org/10.1016/j.future.2020.04.021.

[15] Damgård, I., Pastro, V., Smart, N., Zakarias, S. (2012). Multiparty Computation from Somewhat Homomorphic Encryption. In: Safavi-Naini, R., Canetti, R. (eds) Advances in Cryptology – CRYPTO 2012. CRYPTO 2012. Lecture Notes in Computer Science, vol 7417. Springer, Berlin, Heidelberg. https://doi.org/10.1007/978-3-642-32009-5_38

[16] Keith Bonawitz, Vladimir Ivanov, Ben Kreuter, Antonio Marcedone, H. Brendan McMahan, Sarvar Patel, Daniel Ramage, Aaron Segal, and Karn Seth. 2017. Practical Secure Aggregation for Privacy-Preserving Machine Learning. In Proceedings of the 2017 ACM SIGSAC Conference on Computer and Communications Security (CCS '17). Association for Computing Machinery, New York, NY, USA, 1175–1191. https://doi.org/10.1145/3133956.3133982

A Actively secure solution

What could go wrong in our passively secure solution? We do check our inputs, which are constructed from verified bits. However, other parts of the protocol could be tampered with:

- The input shares are not necessarily consistent. Shamir sharing requires that all shares lie on the same t-degree polynomial.
- We use a passively secure construction for opening a secret-sharing, without checking the consistency of the communicated shares.
- The non-interactive constructions for generating random secrets are passively secure. The shares are not automatically consistent.
- The multiplication protocol from Subsection 2.1 is passively secure. Shares could be modified before revealing the product.
- The outcome of a zero-check is revealed. A party might modify its share (e.g. use different weights), such that the zero-check outcome reveals something about other inputs.

Although we do not completely work out all details, we suggest how to modify our passively secure solution, such that it becomes actively secure with dishonest minority. As usual, this will seriously affect performance, although we tried to find a modestly efficient version, inspired by [5].

Having such an actively secure protocol, all that a dishonest member can achieve, is causing the protocol to abort; the protocol will abort when the honest parties detect cheating. The adversaries will not learn secrets of the honest parties, only the intended output(s) of the protocol. The protocol will be computationally secure, its security depending on the hardness assumption of the PKI.

A.1 Consistent input shares

Assuming we have a construction (see Subsection A.3) to generate a consistent random secret sharing $\langle r\rangle_t$, and a way to securely reconstruct a secret, we can make an actively secure protocol for providing inputs:

1. The parties generate a consistent random secret sharing $\langle r \rangle_t$.
2. They reconstruct r towards input party i.
3. Input party i broadcasts $x_i - r$, x_i being its input.
4. The parties compute $\langle x \rangle_t = (x - r) + \langle r \rangle_t$.

It is important that each party receives the same $x - r$. They need to check this, e.g. by sending a hash of a batch of $x_i - r$'s to each other. This idea of generating consistent input shares is described e.g. in the famous SPDZ paper [15].

A.2 Consistent reconstruction

Suppose the parties have a secret sharing $\langle x \rangle_t$ of a secret x and want to reveal x. A well-known way to reconstruct x with at most t malicious players is:

1. Each party broadcasts its share of x to each other party.
2. Each party checks whether all received shares are consistent, i.e. whether they lie on the same t-degree polynomial.
3. Each party reconstructs x with the public Lagrange coefficients.

The consistency check can be done by reconstructing a polynomial from $t + 1$ shares, and verifying that the remaining shares comply with it.

A.3 Generating random secrets

The idea of generating random secrets without interaction stems from [2]. They describe an actively secure variant for $t < n/4$. To achieve active security with malicious minority ($t < n/2$), we need a different approach.

Suppose the parties want to jointly generate a t-degree secret-sharing $\langle r \rangle_t$ of a random number r that none of the parties is allowed to learn.

1. Each party i generates a random number r_i, creates a secret sharing for it, and distributes the shares to all players.
2. The parties locally add up the received shares, and generate $\langle r \rangle_t = \langle \sum_i r_i \rangle_t$.

This requires quite some communication, so if one needs to generate many random numbers, a more efficient approach is to use a Vandermonde matrix [4]. Furthermore, the generated shares are not guaranteed to be consistent. To check for consistency one needs to open a random linear combination of these secret sharings [6] and sacrifice one.

A.4 Actively secure multiplication

In Subsection 2.1 we locally multiply shares, such that the products lie on the same $2t$ degree polynomial. We need $2t+1$ degrees to reconstruct that polynomial, such that we cannot achieve security with malicious minority. We first need to reduce the polynomial degree to t, and then reconstruct it, so we need another approach.

Given two t-degree Shamir secret-shared numbers $\langle x \rangle_t$ and $\langle y \rangle_t$, the parties jointly compute a t-degree Shamir secret-sharing $\langle z \rangle_t$, such that $z = x \cdot y \pmod{p}$:

1. They locally multiply their shares of x and y, and obtain a $2t$-degree sharing of $z = x \cdot y$.
2. Each party generates a t-degree sharing of its share of z, and distributes the shares to all players.
3. Each party computes the public Lagrange coefficients, and linearly combines the received shares to a t-degree secret-sharing $\langle z \rangle_t$.

This secure multiplication is still passively secure. An adversary could modify his shares, such that the parties output a secret-sharing of $z + d$, instead of z. However, besides this additive attack, the protocol is actively secure, as shown in [7].

To exclude this additive attack, we need to run an additional zero-check (see Subsection A.5) after having computed $\langle z \rangle_t$:

1. The parties generate a secret random number $\langle r \rangle_t$ (see Subsection A.3).
2. They securely multiply $\langle w \rangle_t = \langle r \rangle_t \cdot \langle z \rangle_t$, as above.
3. The parties open r (see Subsection A.2).
4. They compute $\langle \omega \rangle_t = \langle w \rangle_t - r \cdot \langle z \rangle_t$.
5. They run the zero-check protocol on ω.

We need an actively secure zero-check protocol for this.

A.5 Actively secure zero-check

Given a t-degree secret sharing $\langle z \rangle_t$, the parties check whether $z = 0$. It uses the re-sharing approach (with additive attack vulnerability) described in the previous subsection.

1. They generate a secret random number $\langle r \rangle_t$ (see Subsection A.3).
2. They securely multiply $\langle r \rangle_t$ with $\langle z \rangle_t$:
   (a) They use resharing to obtain $\langle r \cdot z \rangle_t$ from $\langle r \cdot z \rangle_{2t}$.
   (b) They generate a secret random number $\langle s \rangle_t$ (see Subsection A.3).
   (c) They use resharing to obtain $\langle w \rangle_t = \langle s \cdot (r \cdot z) \rangle_t$ from $\langle s \cdot (r \cdot z) \rangle_{2t}$.
   (d) They open s and compute $\langle \omega \rangle_t = \langle w \rangle_t - s \cdot \langle r \cdot z \rangle_t$.
   (e) They reconstruct ω and check $\omega = 0$.
3. They open $r \cdot z$.
4. They accept if all parties find that $r \cdot z = 0$.

The opening of ω might leak information on $r \cdot z$, but since r is secret, no information on z is leaked. It is needed to avoid the additive attack in the multiplication of r and z. This protocol is actively secure, with cheating (and failure) probability $1/p$ [7].

B Non-interactively generating a zero-sharing

For security reasons we need to add a zero-sharing when securely multiplying two Shamir secret-sharings, to avoid leakage of the inputs [2]. A straightforward approach would require additional communication between the parties to achieve this. Fortunately, a more elaborate approach is known to do this non-interactively. This approach requires additional machinery: replicated secret-sharing and pseudo-random functions (PRFs). In this appendix we explain the main ideas behind the approach, which are adequately described in the PhD thesis of Sebastiaan de Hoogh [10].

A replicated secret-sharing $\langle s \rangle^R$ of secret s between n parties with threshold t consists of $w = \binom{n}{t}$ random numbers r_i, each i, $1 \leq i \leq w$, corresponding with a different subset τ_i of $\{1, 2, \ldots, n\}$ with cardinality t, such that $s = \sum_{i=1}^{w} r_i$. The idea is that each party i only knows the random numbers r_j for which $i \notin \tau_j$.

The nice thing with replicated secret-sharing is that many random replicated secret-sharings can be interactively generated, given only one. Namely, the parties can locally generate fresh random numbers using a PRF: $r_i \leftarrow \mathrm{PRF}(r_i, i)$, for each r_i they know.

To generate a Shamir secret-sharing of zero of degree $2t$, the parties need to randomly generate a $2t$-degree polynomial $z(x)$, such that $z(0) = 0$, and each party i should be able to compute its share

$z(i)$. This can be achieved by generating t random replicated secret-sharings $\langle r_i \rangle^R$, $1 \leq i \leq w$, and constructing

$$z(x) = \sum_{i=1}^{w} p_i(x)(\langle r_1 \rangle_i^R x + \ldots \langle r_t \rangle_i^R x^t),$$

where polynomial

$$p_i(x) = \prod_{j \in \tau_i} \frac{j - x}{j},$$

and $\langle r_j \rangle_i^R$ denotes the ith random number of replicated secret-sharing $\langle r_j \rangle^R$, corresponding with subset τ_i.

Since the $p_i(x)$ have degree t, indeed polynomial $z(x)$ has degree $2t$. Furthermore, $z(0) = 0$, and each party j can compute $z(j)$, because $p_i(x) = 0$ whenever $j \in \tau_i$ (and party j doesn't know the ith random number). For more details we refer to [10]. For our protocol it is important that by generating only one replicated secret-sharing, the parties can non-interactively generate many $\langle 0 \rangle_{2t}$.
